# Clinical standards for the dosing and management of TB drugs

**DOI:** 10.5588/ijtld.22.0188

**Published:** 2022-06-01

**Authors:** J. W. C. Alffenaar, S. L. Stocker, L. Davies Forsman, A. Garcia-Prats, S. K. Heysell, R. E. Aarnoutse, O. W. Akkerman, A. Aleksa, R. van Altena, W. Arrazola de Oñata, P. K. Bhavani, N. van‘t Boveneind-Vrubleuskaya, A. C. C. Carvalho, R. Centis, J. M. Chakaya, D. M. Cirillo, J. G. Cho, L. D’Ambrosio, M. P. Dalcolmo, P. Denti, K. Dheda, G. J. Fox, A. C. Hesseling, H. Y. Kim, C. U. Köser, B. J. Marais, I. Margineanu, A. G. Märtson, M. Munoz Torrico, H. M. Nataprawira, C. W. M. Ong, R. Otto-Knapp, C. A. Peloquin, D. R. Silva, R. Ruslami, P. Santoso, R. M. Savic, R. Singla, E. M. Svensson, A. Skrahina, D. van Soolingen, S. Srivastava, M. Tadolini, S. Tiberi, T. A. Thomas, Z. F. Udwadia, D. H. Vu, W. Zhang, S. G. Mpagama, T. Schön, G. B. Migliori

**Affiliations:** 1 Sydney Institute for Infectious Diseases, The University of Sydney, Sydney, NSW, Australia; 2 School of Pharmacy, The University of Sydney Faculty of Medicine and Health, Sydney, NSW, Australia; 3 Westmead Hospital, Sydney, NSW, Australia; 4 Department of Clinical Pharmacology and Toxicology, St Vincent’s Hospital, Sydney, NSW, Australia; 5 St Vincent’s Clinical Campus, University of NSW, Kensington, NSW, Australia; 6 Division of Infectious Diseases, Department of Medicine, Karolinska Institutet, Solna, Sweden; 7 Department of Infectious Diseases Karolinska University Hospital, Solna, Sweden; 8 Desmond Tutu TB Centre, Department of Paediatrics and Child Health, Stellenbosch University, Tygerberg, South Africa; 9 Department of Pediatrics, University of Wisconsin, Madison, WI; 10 Division of Infectious Diseases and International Health, University of Virginia, Charlottesville, VA, USA; 11 Department of Pharmacy, Radboud Institute for Health Sciences & Radboudumc Center for Infectious Diseases, Radboud University Medical Center, Nijmegen, The Netherlands; 12 University of Groningen, University Medical Center Groningen, Department of Pulmonary Diseases and Tuberculosis, Groningen, The Netherlands; 13 University of Groningen, University Medical Center Groningen, Tuberculosis Center Beatrixoord, Haren, The Netherlands; 14 Educational Institution “Grodno State Medical University”, Grodno, Belarus; 15 Asian Harm Reduction Network (AHRN) and Medical Action Myanmar (MAM) in Yangon, Myanmar; 16 Belgian Scientific Institute for Public Health (Belgian Lung and Tuberculosis Association), Brussels, Belgium; 17 Indian Council of Medical Research-National Institute for Research in Tuberculosis-International Center for Excellence in Research, Chennai, India; 18 Department of Clinical Pharmacy and Pharmacology, University Medical Center Groningen, University of Groningen, Groningen, The Netherlands; 19 Department of Public Health TB Control, Metropolitan Public Health Services, The Hague, The Netherlands; 20 Laboratório de Inovações em Terapias, Ensino e Bioprodutos (LITEB), Instituto Oswaldo Cruz, Fundação Oswaldo Cruz, Rio de Janeiro, RJ, Brazil; 21 Servizio di Epidemiologia Clinica delle Malattie Respiratorie, Istituti Clinici Scientifici Maugeri Istituto di Ricovero e Cura a Carattere Scientifico (IRCCS), Tradate, Italy; 22 Department of Medicine, Therapeutics and Dermatology, Kenyatta University, Nairobi, Kenya; 23 Department of Clinical Sciences, Liverpool School of Tropical Medicine, Liverpool, UK; 24 Emerging Bacterial Pathogens Unit, Division of Immunology, Transplantation and Infectious Diseases, IRCCS San Raffaele Scientific Institute, Milan, Italy; 25 Parramatta Chest Clinic, Parramatta, NSW, Australia; 26 Public Health Consulting Group, Lugano, Switzerland; 27 Reference Center Hélio Fraga, Fundação Oswaldo Cruz (Fiocruz), Rio de Janeiro, RJ, Brazil; 28 Division of Clinical Pharmacology, Department of Medicine, University of Cape Town, Cape Town, South Africa; 29 Centre for Lung Infection and Immunity, Department of Medicine, Division of Pulmonology and UCT Lung Institute, University of Cape Town, Cape Town, South Africa; 30 University of Cape Town Lung Institute & South African MRC Centre for the Study of Antimicrobial Resistance, Cape Town, South Africa; 31 Faculty of Infectious and Tropical Diseases, Department of Immunology and Infection, London School of Hygiene & Tropical Medicine, London, UK; 32 Faculty of Medicine and Health, Sydney Medical School, The University of Sydney, Sydney, NSW, Australia; 33 Woolcock Institute of Medical Research, Glebe, NSW, Australia; 34 Department of Genetics, University of Cambridge, Cambridge, UK; 35 Department of Infectious Diseases and Microbiology, The Children’s Hospital at Westmead, Westmead, NSW, Australia; 36 Antimicrobial Pharmacodynamics and Therapeutics, Department of Molecular and Clinical Pharmacology, Institute of Translational Medicine, University of Liverpool, Liverpool, UK; 37 Clínica de Tuberculosis, Instituto Nacional de Enfermedades Respiratorias, Ciudad de México, Mexico; 38 Division of Paediatric Respirology, Department of Child Health, Faculty of Medicine, Universitas Padjadjaran, Hasan Sadikin Hospital, Bandung, Indonesia; 39 Infectious Disease Translational Research Programme, Department of Medicine, Yong Loo Lin School of Medicine, National University of Singapore, Singapore; 40 Institute for Health Innovation & Technology (iHealthtech), National University of Singapore, Singapore; 41 Division of Infectious Diseases, Department of Medicine, National University Hospital, Singapore; 42 German Central Committee against Tuberculosis (DZK), Berlin, Germany; 43 Infectious Disease Pharmacokinetics Laboratory, Pharmacotherapy and Translational Research, University of Florida College of Pharmacy, Gainesville, FL, USA; 44 Faculdade de Medicina, Universidade Federal do Rio Grande do Sul, Porto Alegre, RS, Brazil; 45 TB/HIV Research Centre, Faculty of Medicine, Universitas Padjadjaran, Bandung, Indonesia; 46 Department of Biomedical Sciences, Division of Pharmacology and Therapy, Faculty of Medicine, Universitas Padjadjaran, Bandung, Indonesia; 47 Division of Respirology and Critical Care, Department of Internal Medicine, Faculty of Medicine, Universitas Padjadjaran/Hasan Sadikin General Hospital, Bandung, Indonesia; 48 Department of Bioengineering and Therapeutic Sciences, Division of Pulmonary and Critical Care Medicine, Schools of Pharmacy and Medicine, University of California San Francisco, San Francisco, CA, USA; 49 Department of TB & Respiratory Diseases, National Institute of TB & Respiratory Diseases, New Delhi, India; 50 Department of Pharmacy, Uppsala University, Uppsala, Sweden; 51 The Republican Research and Practical Centre for Pulmonology and TB, Minsk, Belarus; 52 National Institute for Public Health and the Environment, TB Reference Laboratory (RIVM), Bilthoven, The Netherlands; 53 Department of Pulmonary Immunology, University of Texas Health Science Center at Tyler, Tyler, TX, USA; 54 Infectious Diseases Unit, IRCCS Azienda Ospedaliero-Universitaria di Bologna, Bologna, Italy; 55 Department of Medical and Surgical Sciences, Alma Mater Studiorum University of Bologna, Bologna, Italy; 56 Blizard Institute, Barts and The London School of Medicine and Dentistry, Queen Mary University of London, London, UK; 57 P. D. Hinduja National Hospital and Medical Research Centre, Mumbai, India; 58 National Drug Information and Adverse Drug Reaction Monitoring Centre, Hanoi University of Pharmacy, Hanoi, Vietnam; 59 Department of Infectious Diseases, National Medical Center for Infectious Diseases, Shanghai Key Laboratory of Infectious Diseases and Biosafety Emergency Response, Huashan Hospital, Shanghai Medical College, Fudan University, Shanghai, People’s Republic of China; 60 Kilimanjaro Christian Medical University College, Moshi, United Republic of Tanzania; 61 Kibong’oto Infectious Diseases Hospital, Sanya Juu, Siha, Kilimanjaro, United Republic of Tanzania; 62 Department of Infectious Diseases, Linköping University Hospital, Linköping, Sweden; 63 Institute of Biomedical and Clinical Sciences, Division of Infection and Inflammation, Linköping University, Linköping, Sweden; 64 Department of Infectious Diseases, Kalmar County Hospital, Kalmar, Linköping University, Linköping, Sweden

**Keywords:** tuberculosis, pharmacokinetics, pharmacodynamics, adverse drug reaction, management, dosing

## Abstract

**BACKGROUND:**

Optimal drug dosing is important to ensure adequate response to treatment, prevent development of drug resistance and reduce drug toxicity. The aim of these clinical standards is to provide guidance on ‘best practice’ for dosing and management of TB drugs.

**METHODS:**

A panel of 57 global experts in the fields of microbiology, pharmacology and TB care were identified; 51 participated in a Delphi process. A 5-point Likert scale was used to score draft standards. The final document represents the broad consensus and was approved by all participants.

**RESULTS:**

Six clinical standards were defined: Standard 1, defining the most appropriate initial dose for TB treatment; Standard 2, identifying patients who may be at risk of sub-optimal drug exposure; Standard 3, identifying patients at risk of developing drug-related toxicity and how best to manage this risk; Standard 4, identifying patients who can benefit from therapeutic drug monitoring (TDM); Standard 5, highlighting education and counselling that should be provided to people initiating TB treatment; and Standard 6, providing essential education for healthcare professionals. In addition, consensus research priorities were identified.

**CONCLUSION:**

This is the first consensus-based Clinical Standards for the dosing and management of TB drugs to guide clinicians and programme managers in planning and implementation of locally appropriate measures for optimal person-centred treatment to improve patient care.

## Abstract (fr)

**CONTEXTE:**

Une posologie optimale est importante afin de garantir une réponse adéquate au traitement, de prévenir le développement de résistances aux médicaments et de réduire la toxicité liée aux médicaments. L’objectif de ces normes cliniques est de donner des indications de « bonne pratique » en matière de posologie et de gestion des agents antituberculeux.

**MÉTHODES:**

Un panel de 57 experts internationaux spécialisés en microbiologie, pharmacologie et soins antituberculeux a été identifié ; 51 ont participé à un processus Delphi. Une échelle de Likert à 5 points a été utilisée pour noter les premières ébauches des normes. Le document final est fondé sur un large consensus puisqu’il a été approuvé par tous les participants.

**RÉSULTATS:**

Six normes cliniques ont été définies : Norme 1, définir la dose initiale la mieux adaptée au traitement de la TB ; Norme 2, identifier les patients potentiellement à risque d’exposition sous-optimale aux médicaments ; Norme 3, identifier les patients à risque de développer une toxicité liée aux médicaments et déterminer comment diminuer au mieux ce risque ; Norme 4, identifier les patients pouvant bénéficier d’un suivi thérapeutique pharmacologique (TDM) ; Norme 5, définir les informations et conseils à fournir aux patients placés sous traitement antituberculeux et Norme 6, enseigner les fondamentaux aux professionnels de santé. Les priorités de recherche ont également été définies, sur la base d’un consensus.

**CONCLUSION:**

Il s’agit des premières normes cliniques, fondées sur un consensus, en matière de posologie et de gestion des antituberculeux. Elles ont pour objectif d’orienter les cliniciens et les responsables de programme en matière de planification et de mise en place de mesures locales adéquates pour un traitement optimal centré sur le patient, afin d’améliorer la prise en charge.

Treatment of TB is aimed at more than simply curing a patient. Possible drug-related adverse effects (AEs) must be balanced against effective treatment to reduce ongoing transmission, prevent future disease, development of drug resistance and chronic post-TB disease. Effective treatment for TB is highly dependent on early diagnosis, and rapid and adequate treatment initiation. Sub-optimal drug exposure often facilitates the emergence of drug-resistant TB, and it is now well-known that drug-related AEs are also common. By studying the absorption, distribution, metabolism and excretion (ADME) of individual TB drugs, as well as the effect of drug transporters,^1^ important differences in pharmacokinetics (PK) have been observed between patients. These inter-individual differences help to explain why some patients show poor treatment response, or have a higher risk of suffering from significant AEs.^2^ The introduction of hollow-fibre infection models has contributed significantly to our understanding of the relation between drug exposure and antibacterial effect.^3^ Detailed dose fractionation studies have identified optimal drug dosing strategies that maximise the treatment efficacy and reduce the risk of acquired drug resistance.^4^ An example of the value of these critical evaluations is our reconsideration of how best to dose rifampicin (RIF),^5^ a drug that has been in clinical use since the 1960s. Initial dosing recommendations were mainly influenced by price considerations and not optimal efficacy. It is now widely known that its maximum therapeutic effect is not achieved at the standard recommended dose,^6^,^7^ a recognised limitation of current treatment regimens. Newer TB drugs such as bedaquiline (BDQ), delamanid (DLM) and pretomanid (Pa),^8^–^10^ as well as repurposed antibiotics (such as moxifloxacin [MFX] and linezolid [LZD]) are widely used in the treatment of drug-resistant TB, but optimal dosing strategies are still being investigated.^11^–^13^

## AIM OF THE CLINICAL STANDARDS

Our aim is to provide guidance on ‘best practice’ for dosing and management of TB drugs, identifying important clinical considerations to inform dosing decisions for both adults and children. For some TB drugs, selecting the most appropriate dose is challenging, as there is limited evidence to inform dose adjustments in specific circumstances. In these situations, the pharmacological principles described should guide dosing decisions.^14^ Fortunately, there is a rapidly growing body of literature informing better TB drug dosing, including in children.^15^

This consensus-based document describes the following activities:

1. Defining the most appropriate initial dose for TB treatment (Standard 1).
2. Identify patients who may be at risk of sub-optimal drug exposure (Standard 2)
3. Identify patients at risk of developing drug-related toxicity and how best to manage this risk (Standard 3).
4. To identify patients who can benefit from TDM (Standard 4).
5. Highlighting education and counselling that should be provided to people initiating TB treatment (Standard 5).
6. Provide essential education for healthcare professionals (Standard 6).

In addition, consensus research priorities were identified.

## METHODS

A panel of global experts was identified to represent the main scientific societies, associations and groups active in global TB management and TB treatment research. Of the 57 experts initially invited, six did not respond after one invitation reminder. All respondents (*n* = 51) were asked to comment via a Delphi process on six draft standards developed by a core team (*n* = 8) of researchers; everyone provided valid answers and constructive input. The final panel included TB clinicians (*n* = 24), TB public health specialists (*n* = 5), TB paediatricians (*n* = 5), pharmacologists (*n* = 12), microbiologists/biologists (*n* = 4) and a TB trials methodologist (*n* = 1). A 5-point Likert scale (5: high agreement; 1: low agreement) was used to indicate agreement with the standards. At the first Delphi round, agreement was high, with a median value of >4.6 (for all standards). Due to the high agreement (defined as quartile deviation [Q3-Q1/2] ≤0.6),^16^ no major changes to the draft standards were made. Based on substantial initial agreement, a draft document was developed by the expert panel. The document underwent two rounds of revisions, and the final version was approved by consensus (100% agreement).

## STANDARD 1

**Every patient should receive the most appropriate drug dose when starting TB treatment to avoid too low or too high drug exposure, which could result in treatment failure or adverse drug effects**

In the current WHO guideline, advice is provided regarding dose adjustment for bodyweight and renal function.^17^ However, other factors contribute to variability in the pharmacokinetics of TB drugs, such as age, malnutrition, hepatic function, diabetes (DM) and HIV status, pregnancy, disease severity, genetic factors predisposing for rapid drug metabolism, drug–drug interactions with concomitant treatment, drug absorption and food-drug interactions. Selecting the most appropriate doses for children is hindered even more by insufficient data on PK, bioavailability and treatment efficacy, especially among the most vulnerable paediatric populations (including those living with HIV, those who are malnourished and those who fall in understudied age groups such as <2 years). These aspects therefore require careful consideration in conjunction with drug susceptibility testing (DST) results (phenotypic and/or genotypic) collected from the patient (or likely source in the context of children) when selecting the optimal treatment regimen and drug dosages to avoid treatment failure and/or AEs.^18^ Details on individual drugs and factors that require consideration are presented in Figure 1.

**Figure:**
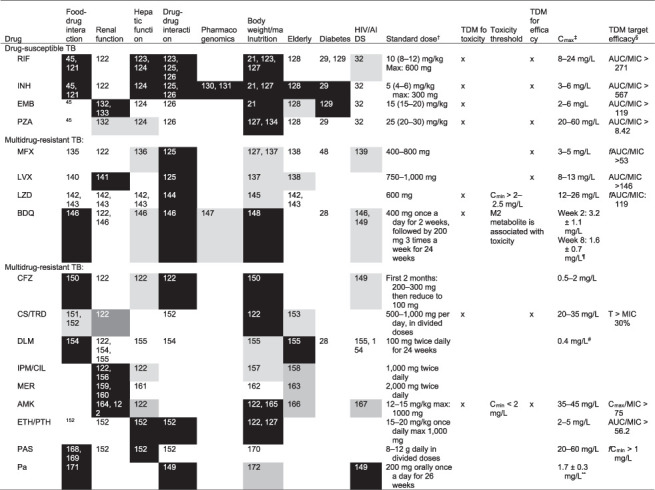
Factors contributing to variability in pharmacokinetics, as well as the efficacy and toxicity of drugs used to treat TB.* *Factors which are likely (black), might (light grey) or are unlikely (white) to contribute to variability in drug efficacy or toxicity and which should be considered when making drug selections or dose adjustments. ^†^WHO-recommended doses for adults.^173^^‡^Reference values for C_max_ after a standard dose.^64^^§^The PK/PD targets were previously reported and are dependent on the precise MIC methodology used in the respective studies.^104^ Because of the systematic differences between some MIC methods, these targets cannot be used directly with some MIC methods.^114^ The PK/PD targets should be used in a multiprofessional team experienced in TDM. ^¶^Reference values for C_max_ after standard dose.^174^^**^Reference value for C_max_ after standard dose.^175^ TDM=therapeutic drug monitoring; C_max_=maximum concentration (of a drug); RIF = rifampicin; AUC = area under the curve; INH = isoniazid; EMB= ethambutol; PZA = pyrazinamide; MFX ¼moxifloxacin; LVX = levofloxacin; LZD = linezolid; BDQ = bedaquiline; CFZ = clofazimine; CS = cycloserine; TRD = terizidone; DLM = delamanid; IPM/CIL = imipenem/cilastatin; MER = meropenem; AMK = amikacin; ETH = ethionamide; PTH = prothionamide; PAS = para-aminosalicylic acid; Pa = pretomanid; MIC = minimum inhibitory concentration; fAUC = free area under the concentration time curve; C_min_ = minimum concentration (of a drug); PK = pharmacokinetics; PD = pharmacodynamics.

Weight-based dose selection of TB drugs should be carefully considered in cases with extremely high or low body weights, and in children, especially when no maximal dose limit is provided. It is important to appreciate that severe states of malnutrition are associated with changes in body composition, hypoproteinaemia, gastrointestinal disturbances (such as diarrhoea and malabsorption) and decreased renal function.^19^,^20^ This can impact the PK of some TB drugs, thereby necessitating dose adjustments.^21^ In children, age-related maturation of drug metabolism pathways may be more important than weight, although the two are usually closely correlated. Age-related effects on renal clearance and metabolism of TB drugs are also present in very young children (<2 years old), often resulting in reduced absorption, metabolism and elimination. A full understanding of the age, weight and potential hormonal impacts on drug metabolism in adolescents is understudied. Increasing age is associated with a decline in renal function, requiring dose adjustment of renally excreted drugs (e.g., amikacin [AMK]). The risk of hepatotoxicity (e.g., for isoniazid [INH], pyrazinamide [PZA]) also increases with age.^22^ Therefore, age and weight must be taken into consideration when deciding on the dose.^23^–^25^

For renally cleared drugs, dose adjustments are required in individuals with renal impairment, including those receiving renal dialysis, to avoid supra-therapeutic drug exposure and the potential for increased risk of AEs.^26^ Similarly, for TB drugs that are predominantly metabolised by the liver or may cause hepatotoxicity, dose adjustments or alternative drugs require consideration in patients with severe hepatic impairment.^26^ In patients with chronic liver disease (e.g., cirrhosis), potentially hepatotoxic drugs should be avoided, particularly if there are alternatives available, given that the risk of severe hepatoxicity and even liver failure is markedly increased.

A risk factor for developing TB disease in case of TB infection,^27^ DM is also associated with delayed treatment response and lower cure rates. The increased risk of relapse and the emergence of drug resistance, particularly in case uncontrolled glucose, are potentially related to altered drug exposure due to delayed gastric emptying and/or drug–drug interactions with hypoglycaemic agents.^28^,^29^ Metformin can be considered a preferred agent as its hypoglycaemic effect was not affected by TB drugs, especially RIF.^30^,^31^ Patients who develop nausea and vomiting while on metformin require alternative treatment.

As to the effect of HIV infection, more than half of the studies that included both HIV-positive and HIV-negative TB groups showed statistically significant alterations in total exposure and/or peak plasma concentrations for at least one first-line TB drug, but studies were too heterogeneous to derive consistent conclusions.^32^ Special attention should be paid to drug–drug interactions.

Pharmacogenomics is known to play an important role in the metabolism of INH, influencing efficacy and risk of AEs.^33^ A meta-analysis demonstrated a 2-fold higher likelihood of bacteriological failure in rapid acetylators compared to slow acetylators.^34^ A randomised controlled trial demonstrated that INH drug-induced liver injury could be prevented in slow acetylators by a dose reduction to 2.5 mg/kg/day and without early treatment failure.^35^ However, determination of NAT-2 genotype (acetylator status) is not routinely available in most settings. Repurposing the widely available GeneXpert (Cepheid, Sunnyvale, CA, USA) platform to perform such tests holds promise for implementation in programmatic care.^36^ The acetylator status can also be assessed by therapeutic drug monitoring (TDM) if this incorporates the measurement of both INH and its metabolite acetyl INH. One single sample of plasma or saliva is enough for the assessment of the concentration ratio (or metabolic ratio) of acetyl INH to INH, which can be translated to fast or slow acetylator status of the patient.^37^–^39^

Rifamycins (RIF, rifapentine [RPT], rifabutin) induce several cytochrome P450 (CYP450) enzymes and drug transporters, which can significantly decrease concentrations of drugs eliminated via these routes.^40^,^41^ Conversely, INH inhibits a range of CYP450 enzymes, including CYP3A4 and therefore can increase concentrations of drugs metabolised by this enzyme.^42^ As the inductive effect of RIF generally outweighs the inhibitory effect of INH, the overall effect is a net decrease in the concentrations of many drugs. Caution should be exercised in the co-administration of nephrotoxic TB drugs (e.g., AMK) with other nephrotoxic drugs (e.g., tenofovir), with regular monitoring of renal function. Older patients, people living with HIV and other immunocompromised patients with TB requiring polypharmacy are at particular risk of drug–drug interactions.

The presence of food can either enhance or reduce drug absorption. As described in the product information, co-administration with food is required for drugs such as BDQ, DLM, Pa, RPT and clofazimine (CFZ), whereas RIF and INH should preferably be taken on an empty stomach,^43^–^45^ but they can be taken with a light meal or snack to prevent or alleviate gastrointestinal AEs and subsequent non-adherence.

When DST demonstrates low-level resistance (LLR), clinicians should consider these findings and, ideally, select a different drug to which the isolate is fully susceptible or increase the dose and use TDM when available. For some drugs, a higher dose can be tolerated and achieve the target exposure (Table 1). Although such a decision can be justified based on pharmacological principles, supportive clinical data are scarce and higher doses to overcome LLR should be avoided where possible. Lower-than-standard doses should be avoided and may only be considered with an otherwise strong core regimen when severe AEs to some drugs, such as LZD, cannot be otherwise avoided or managed.^46^

**Table 1:** Overview of low-level drug resistance mechanisms for key first and second-line TB drugs and their corresponding PK/PD targets for TDM and increased dosing strategies

Drug	Range*	Mode of susceptible MIC distribution*	CC*	LLR mutation(s)^†^	Typical LLR MIC-range*	Standard dose?^‡^	High dose^‡^	Maximum dose^§^	TDM?	Target AUC/MIC^¶^
RIF	0.016–0.25	0.06	0.5	Borderline resistance mutations^#^	0.125–4	No	20–35 mg/kg	2,100 mg	Yes	>271
INH	0.016–0.125	0.06	0.1	*inh*A (c-15t)	0.25–1	No	10 mg/kg	900 mg	Yes	>567
LVX	0.125–1	0.5	1	*gyr*A A90V, S91P and D94A	2–4	No	15–20 mg/kg	1,500 mg	Yes	>146
MFX	0.064–0.25	0.125	0.25	*gyr*A A90V, S91P and D94A	0.125–2	No	10–15 mg/kg	800 mg	Yes	>53

^*^ All figures in mg/L tested using non-standardised protocols as reported in the literature.^109^,^110^ These values apply to MGIT and cannot necessarily be used for other growth media because systematic differences may exist compared with MGIT.^114^

^†^ A higher dose should only be considered, if no additional mutations are present that may raise the MIC even further, thereby conferring high-level resistance (e.g., *kat*G S315T in addition to *inh*A c-15t or *gyr*A D94G in addition to *gyr*A A90V).^109^,^110^,^176^,^177^ Therefore, the detection of high-level resistance mutations or MICs of >1 mg/L for MFX (i.e., the WHO clinical breakpoint) and, >1 mg/L for INH (CLSI currently recommends >0.4 mg/L) are exclusion criteria for the use of these agents, irrespective of the dose used.^109^,^110^,^176^

^‡^ When one of these low-level resistance mutations is present, the standard dose is insufficient and should not be used. The level of evidence for whether, and to what extent, low-level resistance can be overcome with a high dose is very low and largely based on expert opinion.^178^–^180^ The use of high-dose MFX has been endorsed by the WHO to overcome low-level resistance as part of the long individualised regimen by extrapolating data to high-dose GFX, which is being extrapolated further to high-dose LVX in this publication.^110^ Given these uncertainties, increased dosing for low-level resistant isolates should be avoided but may be critical where only less effective or more toxic drugs are available. If higher doses are used in this context, the cautious approach would be to use TDM to verify the drug exposure and not to consider the agent in question as a core drug of the regimen.

^§^ Drug safety at higher dosages is important, active monitoring and use of TDM can help to increase safety.

^¶^ The PK/PD targets were previously reported and are dependent on the precise MIC methodology used in the respective studies.^104^ Because of the systematic differences between some MIC methods, these targets cannot be used directly with other MIC methods.^114^ The PK/PD targets should be used in a multi-professional team experienced in TDM.

^#^ L430P, D435Y, H445L/N/S, L452P, and I491F.^109^

PK = pharmacokinetics; PD = pharmacodynamics; TDM = therapeutic drug monitoring; CC = critical concentration; LLR = low-level resistance; MIC = minimum inhibitory concentration; AUC = area-under the concentration time curve; RIF = rifampicin; INH = isoniazid; LVX = levofloxacin; MFX = moxifloxacin; MGIT = Mycobacteria Growth Indicator Tube; CLSI = Clinical and Laboratory Standards Institute.

## STANDARD 2

**Patients should be re-evaluated when demonstrating slower response to TB treatment than expected**

Further investigation is warranted when patients are showing signs of sub-optimal treatment response, such as lack of clinical improvement or persistent sputum smear or culture positivity (>2 months for drug-susceptible TB), despite phenotypically proven drug susceptibility. Patients should be evaluated for the following risk factors associated with sub-optimal drug exposure: sub-optimal drug dosing, drug–drug interactions, food–drug interactions, gastrointestinal conditions (e.g., malabsorption and diarrhoea), diabetes, HIV/AIDS, non-adherence, host genetic factors predisposing for rapid drug metabolism (e.g., NAT2 genotype), pathogen factors strains with borderline or LLR as determined using phenotypic and/or genotypic DST. As drug penetration in TB cavities can be sub-optimal, verification of drug concentrations and/or higher dosages may be required for adequate treatment response.^47^

Although it is critically important to assess for medication adherence and underlying reasons contributing to potential non-adherence, it is equally important to assess for other factors that could contribute to sub-optimal drug exposure (e.g., the right dose has been prescribed and no drug– or food–drug interactions compromising drug exposure are present). Patients with other medical conditions such as gastrointestinal conditions, DM^48^,^49^ and/or HIV/AIDS^50^ may benefit from TDM, which allows for dose individualisation based on measurement of drug concentrations (Figure 1).^51^,^52^ If these issues have been ruled out or considered unlikely, repeating DST is recommended to exclude any possibility of drug-resistant disease, especially in situations including presence of cavitary disease, and/or prior history of TB treatment, or pregnancy. When DST results are discrepant, appropriate adjustments should be made. If drug susceptibility of the *Mycobacterium tuberculosis* isolate is reconfirmed for a patient who is not responding adequately to treatment, it is important to consider the potential for rapid metabolism of INH.^34^,^35^ Ideally, a pharmacogenetic test or TDM should be performed to determine the acetylator status of the patient.

As drug exposure reflects all these underlying factors, TDM may be recommended in patients not responding to treatment when available (Figure 1).^51^–^54^

## STANDARD 3

**The risk of TB drug toxicity should be minimised by initial screening and ongoing clinical monitoring. Toxicity specific to TB drugs should be prevented and appropriately managed to prevent harm and limit its contribution to poor treatment-adherence**

As a result of receiving a multidrug combination, TB patients may experience various AEs.^55^ Although the global prevalence of AEs is generally underreported, AEs are more commonly experienced (range: 8–96%) than previously appreciated.^56^ AEs include gastrointestinal disturbances, hepatotoxicity, ototoxicity, nephrotoxicity and peripheral neuropathy (Table 2).^56^ Cutaneous reactions (not dose-/concentration-dependent) to first- and second-line TB drugs have also been reported.^57^ Certain AEs can be life-threatening if not identified early and promptly managed.^58^,^59^ Several risk factors, which can be classified as patient, drug and social, contribute to AE risk and require consideration of dose adjustments (Table 2).^60^

**Table 2:** Toxicity associated with TB drugs, potential risk factors for developing drug toxicity and recommended monitoring practices *

Drug	Toxicity	Patient populations at potentially greater risk of toxicity)	Suggested monitoring (frequency)	Comments
Drug-susceptible TB				
INH	Hepatotoxicity Peripheral neuropathy Cutaneous reactions	Older patients (>65 years), sex (male),^181^ diabetes mellitus,^182^ host genetics (*NAT2* variants for isoniazid slow or intermediate acetylators,^131^,^183^,^184^ HIV,^185^ liver disease	Liver function tests, peripheral sensibility (monthly)	To minimise risk of peripheral neuropathy, co-administrated of vitamin B6 is recommended, particularly in patients at risk (e.g., children, during pregnancy or if alcohol abuse or other predisposing condition is present). In patients with liver disease (without cirrhosis) and with AST and ALT >5x ULN, consider an alternative regimen like RIF, EMB and LVX. In patients with cirrhosis, consider use of an alternative regimen (with an injectable, EMB and LVX)
RIF	Hepatotoxicity Gastrointestinal disturbances Cutaneous reactions	Sex (male),^181^ HIV,^185^ liver disease, polypharmacy (drug-drug interactions)	Liver function (monthly)	Check for drug-drug interactions with all accompanying drugs, online data bases available. Educate patients about discoloration of body fluids. In patients with cirrhosis, consider use of an alternative regimen (with CPM, EMB, and LVX)
RPT	Hepatotoxicity Gastrointestinal disturbances	Sex (male),^181^ HIV,^185^ liver disease, polypharmacy (drug-drug interactions)	Liver function (monthly)	Check for drug-drug interactions with all accompanying drugs, online data bases available
PZA	Hepatotoxicity Optic neuritis Nephropathy Gout, joint and muscle pains Gastrointestinal disturbances Cutaneous reactions	Older patients (>65 years), sex (male),^181^ HIV,^185^ liver disease, renal disease	Liver function (monthly)	Watch out for rash, drug-induced liver injury and arthralgia. Omit in older patients (>65 years). In patients with liver disease (without cirrhosis) and with AST and ALT > 5x ULN, consider an alternative regimen with RIF, EMB and LVX. In patients with cirrhosis, consider use of an alternative regimen (with CPM, EMB and LVX). In patients with creatinine clearance <30 mL/min, consider using EMB and PZA only 3 times a week (at the usual dose)
EMB	Ocular neuritis	Children (<2 years),^186^,^187^ diabetes mellitus,^188^ older patients (>65 years), HIV,^185^ renal disease^189^	Colour/visual acuity (monthly)	Reduce or stop with renal insufficiency. Use commonly avoided in young children and patients that cannot reliably report colour/visual acuity
• Multidrug-resistant TB				
FQs (LVX/MFX)	Neurotoxicity QTc prolongation Musculoskeletal Gastrointestinal disturbances	HIV (185), children (<2 years), children and older patients (>65 years) (increased risk of tendon damage)	(Electrolytes), QTcF interval,^1^ painful tendons/joints (monthly)	LVX is less likely to cause QTcF prolongation than MFX.^190^ MFX can cause liver toxicity. LVX needs dose reduction with renal insufficiency Risk of Achilles tendinitis or rupture (especially when combined with corticosteroids), arthralgia and and aortic aneurysm/dissection (rare events). Reduced seizure threshold. *Clostridium difficile* associated diarrhoea
BDQ	QTc prolongation	Children (<2 years),^186^,^187^ HIV^185^	Electrolytes, liver function, QTcF interval (2 weeks, 12 weeks and 24 weeks)	An increased monitoring of baseline, 2 weeks and monthly ECGs during the treatment is recommended if BDQ is used in combination with other QT-prolonging drugs such as FQs and CFZ. Check for drug-drug interactions^191^
LZD	Hepatotoxicity Optic and peripheral neuropathy Myelosuppression	Older patients (>65 years), HIV,^185^ host genetics	Colour/visual acuity, full blood count, peripheral neuropathy (monthly)	Beware of lactic acidosis and serotonin syndrome due to drug interactions
CS/TRD	Neurotoxicity: seizure, headache, lethargy, confusion, mood change, drowsiness, anxiety, psychosis, depression, suicidal ideation	Epilepsy, depression, psychosis, severe anxiety	Mental health evaluation (monthly)	Beware of suicide ideations and peripheral neuropathy. Avoid or monitor combination with INH and thionamides (increased risk of neurotoxicity). Administer concomitantly pyridoxine (vitamin B6)
CFZ	Gastrointestinal disturbances Pink, red or brownish-black discoloration of skin, body fluids and faeces Photosensitivity QTc prolongation	Severe hepatic impairment	Electrolytes, liver function, QTcF interval	Skin discoloration or hyperpigmentation is common and can be disturbing to patients. Take with food to improve bioavailability and gastrointestinal tolerance. Protect skin from the sun
DLM	Ocular toxicity QTc prolongation	Children (<2 years),^186^,^187^ HIV^185^	Electrolytes, liver function, QTcF interval (2 weeks, 12 weeks and 24 weeks)	
Imipenem-cilastatin/meropenem^3^	Neurotoxicity (confusion, seizures)	History of seizures, renal impairment		Used in combination with amoxicillin-clavulanic acid. Beware of LFT rise with meropenem and reduced seizure threshold with imipenem/cilastatin. *Clostridium difficile* associated diarrhoea
AMK	Nephrotoxicity Ototoxicity Electrolyte disturbances	Older patients (>65 years), HIV (185), renal, vestibular, auditory or severe hepatic impairment	Renal and electrolyte function, audiometry (monthly)	TDM for AMK involves trough levels to avoid toxic concentrations and is highly recommended if available.^76^,^92^ Formal hearing testing must be done for children and adults at baseline and at 2 weeks and regularly thereafter at fortnightly or monthly intervals. Avoid in patients that cannot perform a hearing test
ETH/PTH	Hepatotoxicity Gastrointestinal disturbances Endocrine disorders (gynaecomastia, hypothyroidism)	HIV^185^	Liver function, TSH/T4	Monitor combination with CS or TRD (increased risk of seizures) and PAS (increased risk of gastrointestinal disturbances and hypothyroidism). Administer concomitantly pyridoxine (vitamin B6)
PAS	Gastrointestinal disturbances Hypothyroidism Hepatotoxicity		Electrolytes, liver function, TSH/T4	Monitor combination with ETH/PTH (increased risk of hypothyroidism and gastrointestinal disturbances)
Pa	Peripheral neuropathy Gastrointestinal disturbances Hepatotoxicity		Liver function	

* ECG monitoring is recommended using the Fridericia method of QT correction. Check concomitant QTc prolonging drugs if QTcF > 450 ms; STOP all QTc prolonging drugs if QTcF> 500 ms. Sometimes other drugs can be spared to avoid stopping TB treatment.

INH = isoniazid; AST = aspartate transaminase; ALT = alanine aminotransferase; ULN = upper limit of normal; RIF = rifampicin; EMB = ethambutol; LVX = levofloxacin; CPM = capreomycin; RPT = rifapentine; PZA = pyrazinamide; FQ = fluoroquinolone; MFX = moxifloxacin; BDQ = bedaquiline; ECG = electrocardiogram; CFZ = clofazimine; LZD = linezolid; CS = cycloserine; TRD = terizidone; DLM = delamanid; LFT = liver function test; TDM = therapeutic drug monitoring; AMK = amikacin; ETH = ethionamide; PTH = prothionamide; TSH = thyroid stimulating hormone; PAS = para-aminosalicylic acid; Pa = pretomanid.

Patient-related risk factors for AEs include age, sex and whether pregnant or nursing, which contribute to individual variability in PK and associated exposure-dependent AEs. Drug exposure can be affected by ADME, which naturally varies with the extremes of age. For example, the NAT2 enzyme does not seem to fully mature until later childhood.^61^ Older patients exhibit changes in body composition and reduction of renal and liver function, predisposing them to AEs.^62^ Furthermore, comorbidities such as HIV/AIDS, DM, and liver and renal diseases are frequently associated with PK variability and AEs, either through organ-specific changes or drug–drug interactions.^63^,^64^ First-line TB drugs can be used during pregnancy, but safety data to support the use of TB drugs during pregnancy are scarce for second-line drugs.^65^ FQs and BDQ should be used with caution, while ETH and aminoglycosides should be avoided.^66^ Human data on DLM and Pa are lacking. The characteristic AE profiles of individual TB drugs require careful consideration in specific patient populations and dose adjustments, and avoidance of the drug may be required/indicated (Table 2).

The joint effects of excessive alcohol consumption and smoking, especially cigarettes, can increase the frequency of severe AEs, most notably in patients with prior hepatic steatosis or cirrhosis.^67^ Alcohol use during TB treatment has also been associated with peripheral neuropathy, hyperuricaemia and optic neuritis.^68^ In addition to monitoring drug exposure and potential dose adjustment, assessment of potential substance use and the need for specific support for lifestyle improvements should be integral to TB care.^69^ States of malnutrition also increase the risk of toxicity.^70^

Routine monitoring and patient counselling/health education is important during TB treatment to avoid and identify AEs early.^71^,^72^ Careful medical history taking is crucial as AEs are not always volunteered by the patient.^73^ Patients should be reassured that TB treatment is generally safe and AEs can be managed if they occur. Although children tend to experience fewer AEs than adults, monitoring recommendations follow the same principles as in adults (Table 2).^74^

Although nausea is the most common, the most important AE to first-line TB drugs is hepatotoxicity, as it can be life-threatening. Liver function test derangements are often mild and transient but can be severe. Drug-induced liver toxicity has been described for all first-line drugs (i.e., RIF, INH, PZA) apart from EMB.^75^ In addition, all patients/parents/care givers should be informed about the red/orange discoloration of urine and other body fluids by RIF, which is universal and not an AE.

Patient awareness and routine monitoring of visual acuity and colour vision (Ishihara chart) to detect optic neuritis is important if treatment involves EMB or LZD (baseline and monthly checks recommended).^76^ Monitoring for visual changes can be challenging in young children, or critically ill patients, and may not be feasible in many settings or where patients have pre-existing eye disease, especially cataract. Despite known challenges in testing visual changes in young children or critically ill patients, this should still be an objective of routine follow-up, or an alternative agent should be considered.

In currently recommended regimens for multidrug-resistant TB (MDR-TB) treatment, one of the Group A drugs most commonly associated with serious AEs is LZD.^72^ The risk of bone marrow suppression with LZD requires regular full blood count monitoring (baseline, 2 weeks, then monthly). Peripheral neuropathy is commonly associated with long-term use of LZD and less frequently with INH, CS and FQs.^72^,^77^ These possible and serious AEs require patient and care provider awareness, as well as monthly peripheral neuropathy and vision assessment. Peripheral neuropathy in young children can be difficult to diagnose and signs may include refusal to bear weight or complaints of pain or irritability.

Several drugs increase the QTc interval, which when significantly elevated (>500 ms), can increase the risk of heart arrhythmias and even cardiac arrest.^78^ Adults and children alike require regular ECG monitoring (baseline, 2 weeks, monthly) when receiving BDQ, DLM, or when two drugs that prolong the QT interval are being used in combination (such as FQs and CFZ).^71^ Fortunately, BDQ-related QTc prolongation is uncommonly associated with clinically significant outcomes such as torsades de pointes. Drug interactions, electrolyte levels and thyroid function should be checked and if necessary, corrected.

The risk of Achilles tendinitis (mostly presenting with pain along the tendon or back of the heel) and rupture, as well as aortic aneurysm/dissection (although uncommon) is important and should be considered with long-term FQ use.^79^ Despite fear of bone and joint abnormalities in children treated with FQs, a growing body of literature indicate no major safety concerns in this population.^80^

Depression with suicidal thoughts and other psychiatric illnesses might be associated with TB disease itself, but it is a particular concern with CS/terizidone use, and has also been reported with INH treatment. Regular review, support and counselling is recommended, especially in at-risk groups, not only at the time of diagnosis, but also throughout treatment.^81^

## STANDARD 4

**Patients can benefit from TDM in specific situations for specific drugs using resource-and setting-appropriate assays**

TDM is intended to detect patients with sub- or supra-therapeutic (potentially toxic) concentrations (Figure 1). TDM should be considered for people at highest risk of PK variability, with clinical conditions in which PK variability carries serious consequences, and for drugs which make up the backbone of multidrug regimens or for which the therapeutic window is narrow.^53^,^82^ The implementation of TDM can be tailored for specific TB services making use of various types of assays (e.g., high-performance liquid chromatography-ultraviolet, liquid chromatography–mass spectrometry or nanophotometer).^54^,^83^ Dose changes guided by TDM should take other clinical parameters into consideration (e.g., severe cavitary disease).^47^ High priority populations to consider for TDM include those with HIV co-infection, DM, malnutrition, or children, because these factors increase the probability of pharmacokinetic variability and are independently associated with poor TB treatment outcomes (Figure 1). In many TB-endemic settings, these conditions frequently overlap.^84^ In malnourished children with TB, sub-therapeutic exposure has been demonstrated despite patients receiving WHO-recommended doses.^85^ Furthermore, PK variability and sub-therapeutic exposures are likely exacerbated by a concurrent enteropathogen burden, which can present additional challenges for TB eradication.^86^

Both DM and/or HIV co-infection conditions predispose patients to malabsorption or delayed drug absorption, depending on the stage of treatment or disease severity, but also represent a priority situation for TDM given the potential for drug–drug interactions.^50^,^87^,^88^ Certain programmatic settings have adopted routine TDM for people with HIV^47^ and DM who initiate TB treatment, and have found that frequent dose adjustments are required to achieve timely microbiological cure.^89^

In patients with central nervous system TB, cerebrospinal fluid concentrations of RIF are only a small fraction of exposure in the serum, and TDM should be routinely performed. High-dose intravenous and oral RIF combined with TDM can be used to target high exposure in serum,^90^ and thereby increase concentrations in the cerebrospinal fluid.

Specific drugs to prioritise for TDM are show in Figure 1. For certain drugs, such as LZD, measurement of the trough concentration is important to mitigate AEs such a mitochondrial toxicity associated with myelosuppression and neuropathy.^11^,^46^,^91^ In the uncommon scenario where AMK is used, TDM should be used to avoid ototoxicity and nephrotoxicity.^92^

Although TDM is currently not readily available in several settings, new developments will facilitate broader implementation.^83^ Modifications to adjust to different settings include investment in high-throughput equipment, such as mass spectrometry, and human expertise at a central level, bypassing cold chain requirements with microsampling techniques such as dried blood spots (DBS)^93^ and volumetric absorptive microsampling, but also the utilisation of currently experimental matrices such as saliva- and urine-based point-of-care testing.^94^–^97^ As TDM technology becomes more readily available, costs are becoming more affordable for resource-limited settings, and with the rise of digital health technologies, access to experts can be more readily facilitated.

## STANDARD 5

**Each patient should undergo counselling/health education regarding their TB treatment and potential adverse effects to improve treatment results, organised according to feasibility and cost-effectiveness criteria, based on the local organisation of health services and tailored to the individual patient’s needs**

A patient-centred approach is an important pillar of the WHO’s End TB Strategy. A key aspect of this approach is to provide counselling and education to all TB patients as described in the Clinical Standards.^71^,^98^ AEs are a frequent cause of poor adherence, treatment interruption and loss to follow-up.^99^ Patients who are aware of potential AEs may be more likely to notify their healthcare team, facilitating more timely management of AEs, potentially reducing the severity of AEs and preventing unfavourable outcomes.^100^ Early consultation with the TB healthcare team may also allow symptomatic treatment of some AEs, such as nausea or skin rash, and reassurance that such effects often improve as treatment progresses.

The initial education of patients (or parents of children with TB) should include information focussed on the prescribed TB medications and the most common AEs, as well as less common, but more severe or important AEs. Anticipatory guidance about the natural history of these AEs could be shared, such as that many AEs will resolve or substantially improve after the first 1–2 weeks of treatment. Patients should also be provided with specific guidance about when to contact the healthcare team and who to contact. As AEs pose a significant challenge to uninformed patients and care givers, which could lead a to drop-off in treatment, education about AEs should occur on a regular basis, within a trusting relationship with the patient and as a component of comprehensive psychosocial support.^101^

## STANDARD 6

**Education for healthcare professionals is important when applying tailored dosing to better understand the link between clinical condition and drug exposure. Additional technical education is required when TDM is used to ensure the quality of the procedure; this includes sampling requirements, drug exposure targets and how to adjust the dose based on drug concentrations**

As TB management is delivered by multidisciplinary teams that include physicians, nurses, counsellors, clinical pharmacologists, laboratory staff, and clinical microbiologists, professional education is required for personalised dosing based on clinical pharmacological principles. Education should include 1) an understanding of how clinical conditions can influence drug exposure, more specifically, how ADME is impacted and how this translates to drug exposure, which should also consider factors such as comorbidities, drug–drug interactions and disease severity;^26^ 2) how the dose-exposure and pathogen susceptibility relate to treatment outcomes (PK and pharmacodynamics [PD] targets) and AEs for balancing efficacy against potential toxicity for prioritised drugs (RIF, INH, PZA, FQs and LZD), including when a “personalised dose” should be considered;^53^,^102^,^103^ and finally, 3) adjustment of dosing based on drug concentrations (TDM) needs to be understood in relation to sampling requirements, including limited sampling schedules.^53^,^103^,^104^ Such training is currently mainly available in specialised TDM centres in high-resource areas and should be expanded for all settings considering or already using TDM.^105^,^106^

Specialised TB nurses are essential for TDM and training is required to ensure appropriate timing of blood samples in relation to drug intake and transport logistics, as well as monitoring of AEs. For clinical pharmacologists and pharmacists there is a need for training in TB pathogenesis in relation to PK/PD targets, as the lengthy treatment duration and characteristics of the disease differs from other pathogens where TDM is applied. A close collaboration within laboratory units is critical, as trained laboratory staff are essential to establish drug concentrations assays. Furthermore, there is a need for education in the application of validation and quality control programmes,^107^ sample stability and transportation requirements, as well as point-of-care testing and the use of alternative sample matrices, such as DBS, other microsampling techniques, saliva or urine.^103^ Clinical microbiologists at TB laboratories should be trained to provide genotypic and/or phenotypic DST for use in TDM,^108^ including guidance of standard vs. high dose assisted by quality-assured MIC determination.^109^–^111^

The resources and healthcare level affect how educational efforts should be structured.^103^ At the community level, a dedicated team is needed to understand how to apply personalised dosing based on screening assays for key drugs to determine low, normal and high drug exposure. The regional level should be trained to support local teams on difficult cases and provide basic training, as well as quantitative assays for individualised dosing. Finally, the central level should be able to provide training modules for other levels, a quality assurance programme^107^ and advanced quantitative assays,^112^ including multiple sample matrices (blood, DBS, saliva, urine) to facilitate the analysis of samples by mail from rural areas or other outpatient settings and also provide dosing software to calculate the drug dose for optimal exposure.^54^

## PRIORITIES FOR FUTURE RESEARCH

Efficacious and safe TB medication will always be a priority for future research. This is especially important for effective but toxic drugs such as LZD, which would benefit from being replaced by a less toxic derivative.^113^ High-quality MIC (using a method that is calibrated against the European Committee on Antimicrobial Susceptibility Testing reference method) and PK/PD data need to be collected and correlated with clinical outcome data during clinical trials to set appropriate breakpoints for phenotypic DST and define PK/PD targets that can be subsequently used during TDM.^114^ This has not been done sufficiently to date, resulting in an incomplete understanding of the mode of action of TB agents. Moreover, operational research can contribute essential evidence on drug dosing in special patient populations including adolescents, pregnant women, malnourished patients and the elderly, as these patients tend to be excluded from Phase 3 clinical trials. Easy-to-use assays facilitating TDM in regional TB clinics and health centres that allow a rapid turnaround time will contribute to better patient management. Cost-effectiveness studies are particularly important in the context of high-burden settings with limited resources, to convince programme managers to offer TDM as part of programmatic care for selected patients without additional costs.

Physicians, pharmacists and other healthcare professionals are encouraged to adequately document personalised dosing practices and impact on treatment.^53^ Evaluation of clinical programmes results in better management of TB treatment.^115^–^117^ When redesigning a TB register, it is recommended to capture data on personalised dosing and TDM, to include a set of core variables which are essential to describe, measure and evaluate the cascade of care in adults, as well as children.^98^ Individual data are preferred over aggregated data, but this depends on the local arrangements. As systematic evaluation of drug dosing, as well as of AEs, is relatively easy with the implementation of computerised, individual registers, this should be considered. This analysis should be included in the annual TB report compiled by TB programmes in many countries.

New drugs (BDQ, DLM, Pa)^115^,^116^ should be included in active drug safety monitoring data to identify AEs of concern, which should be reported to regulatory authorities and the WHO.

Specific attention is needed for children, as child-friendly formulations are not always available for all drugs and in all countries.^98^,^118^ Formulation can have substantial effects on PK and thus safety and efficacy, as well as on palatability.^119^,^120^ Appropriate use of the available child-friendly formulations, and recording the formulation used where feasible, would contribute useful information.

Data protection laws and other restrictions at the country or regional level may limit the type of data that can be collected and may necessitate amending modalities of data collection and storage. Although the long-term follow-up of patients is not considered feasible,^98^ if, for any reason patients, are followed up after treatment completion for rehabilitation or research purposes, any change of status (e.g., TB recurrence) or long-term AEs need to be notified to health authorities to update the TB register.^98^

## CONCLUSION

Programmatic TB treatment has saved many lives and is suitable for most patients, but careful risk stratification is warranted, and certain patients would benefit from a more person-centred approach, for example, by tailoring the optimal drug dose needs to relevant patient features, DST results, the drugs required for effective treatment, as well as the local environment and available resources. The Clinical Standards articulated here are intended to ensure that TB treatment is safe and effective in every single patient.
